# The Correlation Between Intra-Abdominal Pressure and Tolerance to Postoperative Hyperthermic Intraperitoneal Chemotherapy for Pseudomyxoma Peritonei

**DOI:** 10.3389/fsurg.2022.797811

**Published:** 2022-02-25

**Authors:** Junye Yu, Lifei Yu, Lin Su, Ying Shi

**Affiliations:** ^1^Department of Nursing, Aerospace Center Hospital, Beijing, China; ^2^Department of Myxoma, Aerospace Center Hospital, Beijing, China; ^3^Surgical Ward 3, Aerospace Center Hospital, Beijing, China; ^4^Medical Ward 1, Aerospace Center Hospital, Beijing, China

**Keywords:** pseudomyxoma peritonei, intra-abdominal pressure, postoperative hyperthermic intraperitoneal chemotherapy, comfort assessment, surgical oncology

## Abstract

**Objective:**

To evaluate the correlation between pain intensity and comfort level in patients with pseudomyxoma peritoneum (PMP) before and after hyperthermic intraperitoneal chemotherapy (HIPEC).

**Methods:**

From June 2018 to June 2019, patients who underwent HIPEC for PMP after surgical treatment in our institute were selected. The intra-abdominal pressure (IAP) and pain intensity (PI) before and after HIPEC were recorded, and the correlation between them was analyzed.

**Results:**

Seventy-four patients received HIPEC 253 times. IAP and PI were significantly higher after perfusion than before perfusion (*P* < 0.05). When IAP < 12 cmH_2_O, the change of PI was not correlated to the increase of IAP, and the patient tolerated the treatment. However, when IAP > 12 cmH_2_O, the increase of PI was significantly associated with IAP and cause significant discomfort during the treatment.

**Conclusion:**

IAP may be a monitoring index for the comfort of PMP patients during the postoperative HIPEC treatment.

## Introduction

Pseudomyxoma peritonei (PMP) is caused by peritoneal implantation of mucinous tumors and has an estimated incidence of 0.2 per 100,000 patients per year (1). The main treatment for PMP is repeated surgical debulking procedures. However, ~76–91% of patients develop recurrence after surgery and show a median survival rate of <5 years (2, 3). Mucinous tumors rarely show distant metastasis because of the “peritoneum-plasma” barrier, which confines the tumors to the abdominal cavity. Therefore, local application of intraperitoneal chemotherapy is more effective compared with systemic chemotherapy.

Intraperitoneal chemotherapy, namely hyperthermic intraperitoneal chemotherapy (HIPEC), is performed using a special pump to increase local tissue drug concentration and antiblastic drug activity in the form of the heating bath after complete cytoreductive surgery (CRS) (4). Through the synergistic effect of local intraperitoneal chemotherapy and hyperthermia, HIPEC increases the sensitivity of tumor cells to chemotherapeutic drugs, thereby maximizing the killing of tumor cells in the abdominal cavity. In addition, the concentration of higher drug concentration into the blood was very low, and few systemic adverse reactions are observed. This method is not only safe but also greatly improves the long-term survival rate of PMP patients. The 10-year survival rate of mucinous tumor patients treated with HIPEC combined with CRS was 54–70% with a recurrence rate of only 24.2% (5–7). These findings confirmed the efficacy of HIPEC in the treatment of PMP (8–12).

Previous studies mainly focused on the increase in intra-abdominal pressure (IAP) to improve the penetration depth and treatment efficiency of intraperitoneal chemotherapy drugs (13, 14). Few studies have examined changes in the IAP increase–inducing pain intensity (PI). Therefore, in this study, we explored the change of PI in PMP patients before and after HIPEC treatment and the association between IAP and PI during HIPEC treatment. Our results may help establish an objective quantitative basis to adjust patient comfort level in subsequent HIPEC treatment.

## Participants and Methods

### Participant Selection

Seventy-four PMP patients who had been hospitalized in our institute from June 2018 to June 2019 were selected. Inclusion criteria of patients were as follows: (1) diagnosed with PMP; (2) underwent surgical treatment; and (3) received postoperative HIPEC treatment. Exclusion criteria were defined as: (1) patients were younger than 20 years old or older than 75 years old; (2) development of neurogenic bladder or past bladder surgery and unable to receive IAP measurement; (3) during HIPEC, the patients had unsuccessful perfusion and stable circulation; (4) the patients used painkillers continuously or used painkillers other than flurbiprofen axetil during perfusion; (5) patients reported frequent coughing or restlessness that made it impossible to measure IAP; and (6) patients received infusion with oxaliplatin, elemene, and other drugs that may cause irritation and pain. The experimental protocol was established according to the ethical guidelines of the Helsinki Declaration and was approved by the Ethics Committee of Aerospace Center Hospital (No.20161228-YN-02). Informed consent was obtained from all participants.

### Postoperative HIPEC

All patients who received bedside HIPEC were placed in the supine position; before perfusion was initiated, patients were routinely given 50 mg flurbiprofen intravenously. The patients began HIPEC treatment 48 h after operation through a thermochemotherapy perfusion machine (Jilin Maida, model rhl-2000b) connected to the abdominal drainage tube retained in advance during operation for the circulation pathway. The speed was controlled at 300–600 ml/min, and the temperature of the entry and exit were controlled at 43.5° and 41°C, respectively. The liquid level in the external liquid storage bag does not decrease during the circulation process as a stable circulation. The treatment time was 60 min after the circulation was stabilized.

### Observation Index and Measurement

IAP and PI of patients were recorded before the beginning of perfusion and <30 min later, when the abdominal perfusion velocity was close to the maximum value. The tolerance level of patients was reflected by the degree of PI. If the patient developed chest tightness, suffocation, nausea, vomiting, and other discomfort symptoms during treatment, corresponding records should be taken thoroughly.

### IAP

Before March 2019, the intra-abdominal temperature measurement catheter was not routinely indwelled, and thus IAP was measured indirectly by a double-lumen Foley catheter in the emptied bladder preoperatively and connected to the urodynamic monitor (Model OT-UD-II, Beijing Wanshengrenhe). IAP was measured after injecting 25 ml of sterile 0.9% sodium chloride at 37–40°C into the bladder through the urinary tube at a rate of 10 ml/min. After March 2019, IAP was measured directly by connecting the pressure measurement device to the temperature measurement urinary tube (8F) placed in the abdominal cavity. For the direct IAP measurement, at 3 min before the measurement, the upper edge of the pubic symphysis was first set as zero and then the abdominal vibration test was performed with a showing positive result; the value was recorded after the instrument showed stable data and the number stopped fluctuating. IAP was represented as cmH_2_O.

### Comfort

In HIPEC, patients have reported abdominal pain, abdominal distension, dyspnea, nausea, and vomiting. Among these events, abdominal pain is the most common and most important indicator for defining whether the patient can tolerate HIPEC perfusion. We used the visual analogous scale (VAS) to assess abdominal pain intensity with a score ranging from 0 to 10, which corresponds to no pain to the worst possible pain.

### Statistical Methods

SPSS 25.0 statistical software was used for data statistical analysis. The measurement data were analyzed by *t*-test or rank-sum test according to the distribution, while the count data were analyzed by χ^2^ test. Logistic regression and the Spearman test were used for correlation analysis. *P* < 0.05 was considered statistically significant.

## Results

### Patient Information

A total of 74 patients were selected for this study; the patients underwent PHIPEC 253 times. The study group included 22 males and 52 females with a median patient age of 60 years. During the perfusion, three patients developed minor adverse events of mild chest tightness, breathless discomfort, and nausea, with an incidence rate of 1.2%. No serious complications occurred in these patients.

### Comparison of Abdominal Measurement and Bladder Measurement

**Figure 1:**
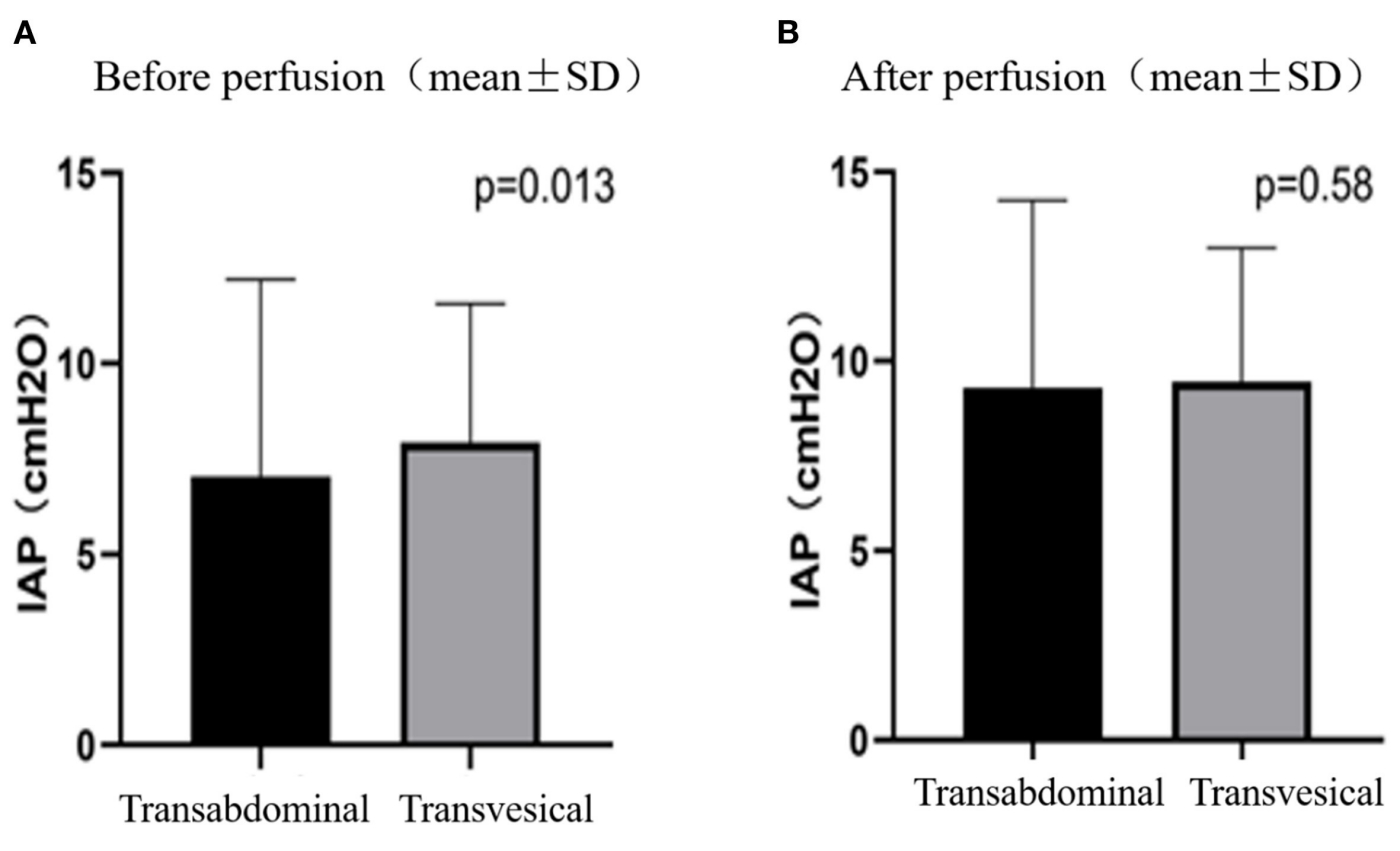
IAPs measured by transabdominal and transvesical method before **(A)** and after **(B)** perfusion.

The patients received intravesical pressure measurement 33 times and transabdominal pressure measurement 220 times. Before HIPEC perfusion, the transabdominal pressure measurement (3.94 ± 3.7 cmH_2_O) was significantly lower than the bladder pressure measurement (5.64 ± 3.1 cmH_2_O) (*P* = 0.013) (Figure 1A). After the perfusion, the transabdominal pressure was measured as 8.25 ± 5.2 cmH_2_O and the bladder pressure as 8.76 ± 4.1 cmH_2_O. There was no significant difference between them (*P* = 0.589) (Figure 1B).

### IAP and PI Before and After Perfusion

**Figure 2:**
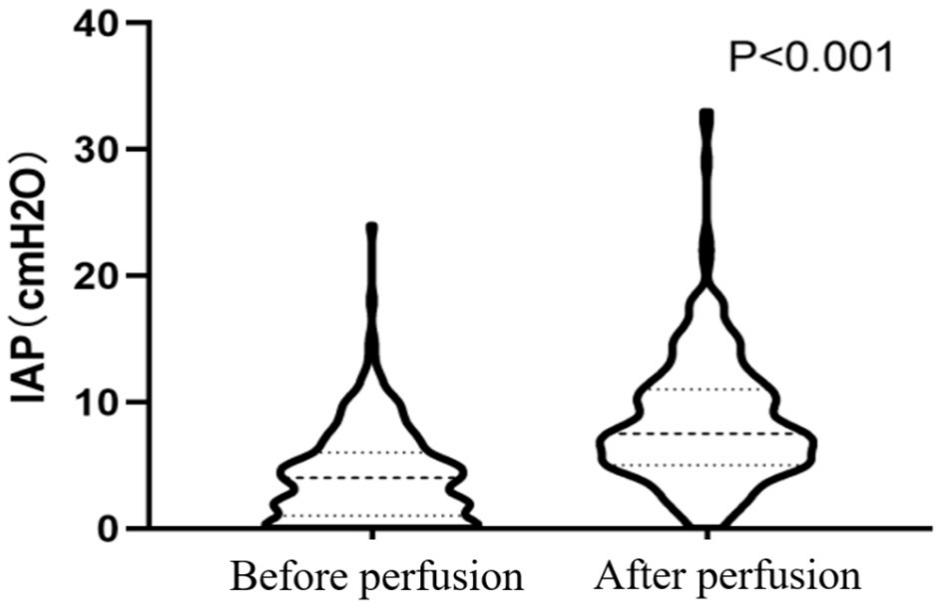
Pressure distribution in the abdominal cavity before and after perfusion.

**Figure 3:**
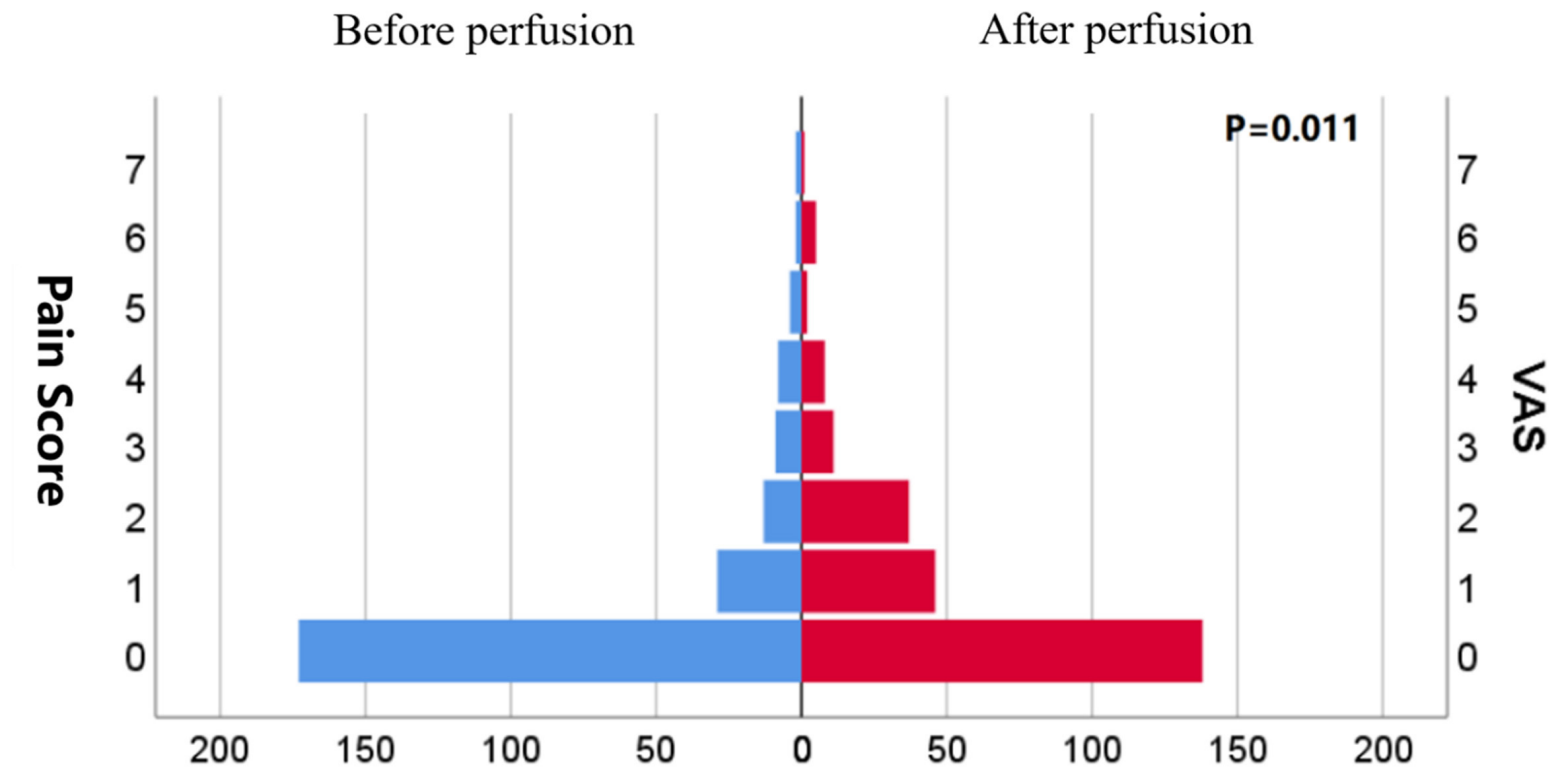
Distribution of pain score before and after perfusion.

The mean IAP after perfusion was 8.32 ± 5.03 cmH_2_O, which was significantly higher than that before perfusion (4.17 ± 3.67 cmH_2_O, *p* < 0.001), and increased by 4.14 ± 4.07 cmH_2_O (Figure 2). The pain score after the perfusion was significantly higher than that before perfusion (*p* = 0.011) (Figure 3).

### Correlation Between IAP and PI

**Figure 4:**
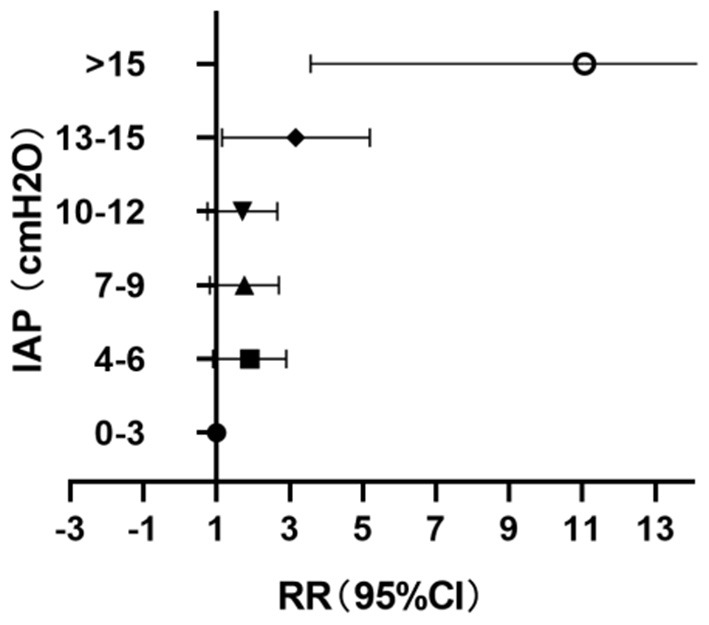
Forest diagram of the relationship between IAP and PI after perfusion.

The values of IAP after the perfusion were stratified every 3 cmH_2_O; 12.7% (32/253) of the patients had IAP <4 cmH_2_O during perfusion and 7.9% (20/253) had IAP > 15 cmH_2_O (Table 1). When IAP ≤ 12 cmH_2_O, the changes of PI with the increase of IAP were not obvious, but when IAP > 12 cmH_2_O, the PI increase significantly correlated with the increase of IAP (*P* = 0.003, RR = 2.74, 95% CI: 1.40–5.37) (Figure 4).

**Table 1:** Differences in pain intensity (PI) in patients with different intra-abdominal pressure (IAP).

**IAP (cmH2O)**	**0–3**	**4–6**	**7–9**	**10–12**	**13–15**	**>15**
***n***	32	65	63	48	25	20
**Lower limit**	1.00	1.00	0.92	0.86	1.40	4.56
**RR**	1.00	1.73	1.60	1.53	2.74	9.37
**Upper limit**	1.00	2.99	2.78	2.74	5.37	19.25
***p***	–	0.05	0.1	0.15	0.003	<0.001

## Discussion

Postoperative HIPEC is a salvage approach to manage peritoneal implantation of mucinous tumors. Since the treatment efficacy and tolerance toward HIPEC depends on IAP, which both contributes to improving the penetration depth of intraperitoneal chemotherapy drugs (13, 14) and also causes significant discomfort in patients. The IAP should thus be carefully monitored during the HIPEC treatment period. Previous studies have shown that the intravesical pressure measurement has good consistency with the real pressure value in the abdominal cavity, which had been the standard method for clinical IAP measurement (15). However, in this study, while direct intraperitoneal pressure measurement before HIPEC perfusion was significantly lower than that of intravesical pressure measurement, after treatment, the two measurements were consistent with each other. These results may be from the abdominal drainage tube being surrounded by fiber cords or intestinal tubes in the abdominal cavity, which obstructs direct pressure measurement in the abdominal cavity, resulting in low measured values. After the abdominal cavity was perfused and circulated with local chemotherapy solution, the patency of the abdominal catheter was kept open and led to consistency with the pressure in the bladder.

This study also showed that HIPEC perfusion simultaneously increased both IAP and PI. A large amount of chemotherapy fluid was gushed into the abdominal cavity during HIPEC treatment to establish the circulatory pathway. Following the expert consensus of China (2019 edition) on the clinical application of intraperitoneal thermal perfusion chemotherapy technology, the volume of perfusion fluid generally can be between 4 and 6 L with the perfusion speed of 400–600 ml/min (16). A large amount of perfusion fluid entered the abdominal cavity in a short time, which led to the increase of intraperitoneal pressure. Clinically, intraoperative HIPEC should be performed under anesthesia to ensure patient tolerance. However, in reality, postoperative HIPEC is performed on conscious patients, and these patients report abdominal pain in that IAP is significantly elevated after a large amount of liquid suddenly distends the abdominal cavity.

In our study, all patients had received painkillers before HIPEC perfusion and successfully completed HIPEC treatment, but the PI of the abdomen increased significantly after treatment, especially in patients who had IAP exceeding 12 cmH_2_O during perfusion (17.5%). In addition, although all patients were able to maintain circulation during clinical perfusion, 12.7% of patients showed low IAP (<4 cmH_2_O) during perfusion, indicating that there might be insufficient intraperitoneal filling and decreased perfusion efficiency. Furthermore, 7.9% of the patients in this study had IAP above 15 cmH_2_O (11 mmHg), which reached the highest limitation for abdominal pressure proposed by the World Society of the Absolute Comparison Syndrome (WSACS), which defined IAP ≥ 12 mmHg as intra-abdominal hypertension (IAH) (17). IAH may cause visceral organ dysfunction; when IAP > 20 mmHg, it may even lead to abdominal compartment syndrome (ACS) and irreversible adverse outcomes (17).

In terms of clinical treatment, higher intraperitoneal pressure contributes to higher tissue penetration and intracellular concentration of chemotherapy drugs in tumor cells, leading to a more efficient treatment effect. However, the increase of IAP may distend the abdomen and subsequently lead to aggravation of abdominal pain and impairment of abdominal visceral function, thereby leading to the decline of tolerance. Strikingly, an increase in IAP even became one of the independent risk factors of patient death (18). Thus, it is particularly important to optimize IAP for satisfying the clinical treatment effect and ensuring the safety of patients. Kong et al. (19) reported that intraperitoneal fluid was 3,000 ml IAP can ensure the success of perfusion fluid circulation when IAP was 4–6 cmH_2_O. However, other researchers used intraperitoneal aerosol chemotherapy to increase IAP to 12~15 mmHg as a treatment scheme for peritoneal tumors (20). In our study, when IAP < 12 cmH_2_O, the symptoms of abdominal pain and abdominal distention in patients treated with HIPEC did not change significantly with the increase of pressure; however, when IAP ≥ 12 cmH_2_O, the degree of abdominal pain and discomfort increased significantly with the increase of pressure. Therefore, we suggest that 12 cmH_2_O should be used as the critical value for monitoring of IAP bedside on conscious patients. In such an IAP setting, the best clinical tolerance can be reached, and the amount of intraperitoneal fluid should be appropriately increased to meet the needs of HIPEC treatment.

## Strengths and Limitations

This study evaluated the discomfort degree of patients with abdominal pain through VAS and the correlation between IAP and PI. Our results help provide an objective quantitative basis for adjusting patient comfort in the follow-up treatment process. This study has several limitations. First, all patients included in this study were at stable circulatory perfusion, which failed to reflect the factors affecting perfusion efficiency. Second, this study was a retrospective study with a limited number of study participants. Therefore, randomized controlled studies are required to verify the relationship between IAP and PI.

## Conclusion

IAP and PI after perfusion are significantly higher than those before perfusion. When IAP < 12 cmH_2_O, the change of PI was not significant with the increase of IAP; however, when IAP >12 cmH_2_O, the PI increases significantly with the increase of IAP. More importantly, when IAP is optimized below 12 cmH_2_O, the patient is well-tolerated under such a setting.

## Data Availability Statement

The raw data supporting the conclusions of this article will be made available by the authors, without undue reservation.

## Ethics Statement

The studies involving human participants were reviewed and approved by the experimental protocol were established, according to the ethical guidelines of the Helsinki Declaration and was approved by Ethics Committee of Aerospace Center Hospital (No.20161228-YN-02). The patients/participants provided their written informed consent to participate in this study.

## Author Contributions

LY: conceptualization, methodology, project administration, validation, data curation, and writing—original draft. JY: validation, supervision, writing—review and editing. LS: resources, data curation, and formal analysis. YS: data curation and visualization. All authors contributed to the article and approved the submitted version.

## Conflict of Interest

The authors declare that the research was conducted in the absence of any commercial or financial relationships that could be construed as a potential conflict of interest.

## Publisher's Note

All claims expressed in this article are solely those of the authors and do not necessarily represent those of their affiliated organizations, or those of the publisher, the editors and the reviewers. Any product that may be evaluated in this article, or claim that may be made by its manufacturer, is not guaranteed or endorsed by the publisher.
